# Supplementary material for: Escherichia coli O157:H7 F9 Fimbriae Recognize Plant Xyloglucan and Elicit a Response in Arabidopsis thaliana
Source: Int J Mol Sci. 2020 Dec 19;21(24):9720. doi: 10.3390/ijms21249720 (PMC7766294; doi:10.3390/ijms21249720)
Supplement: Supplementary file 1 [file ijms-21-09720-s001.zip › ijms-1008624 supplementary Figure.pdf]

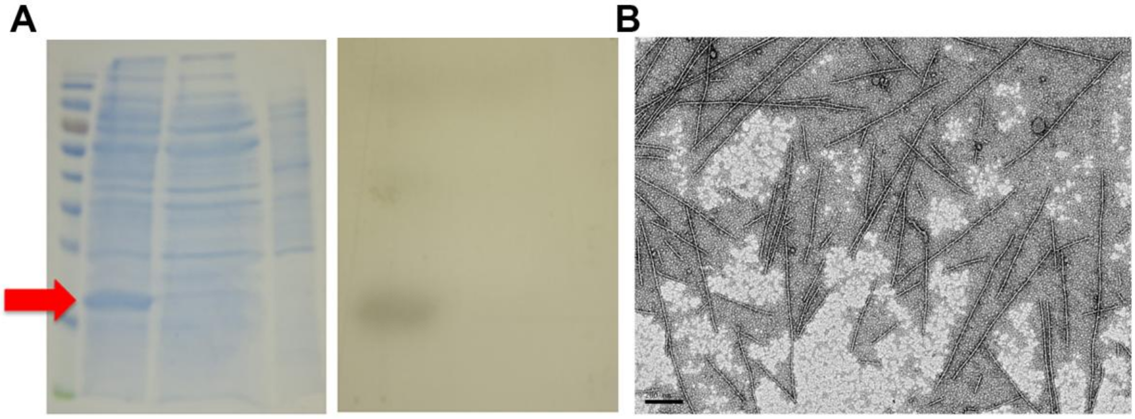

Figure S1. F9 fimbriae antibody specificity and electron micrograph of purified F9 fimbriae.

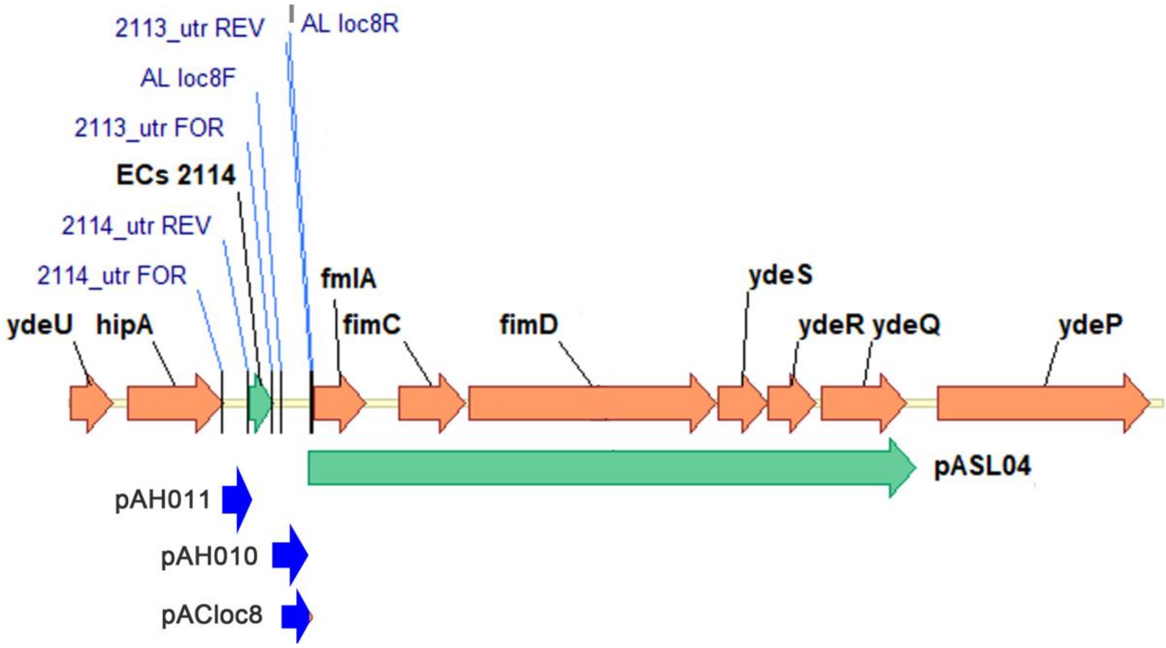

Figure S2. EHEC (Sakai) F9 fimbriae gene organization and reporter constructs used in this study.

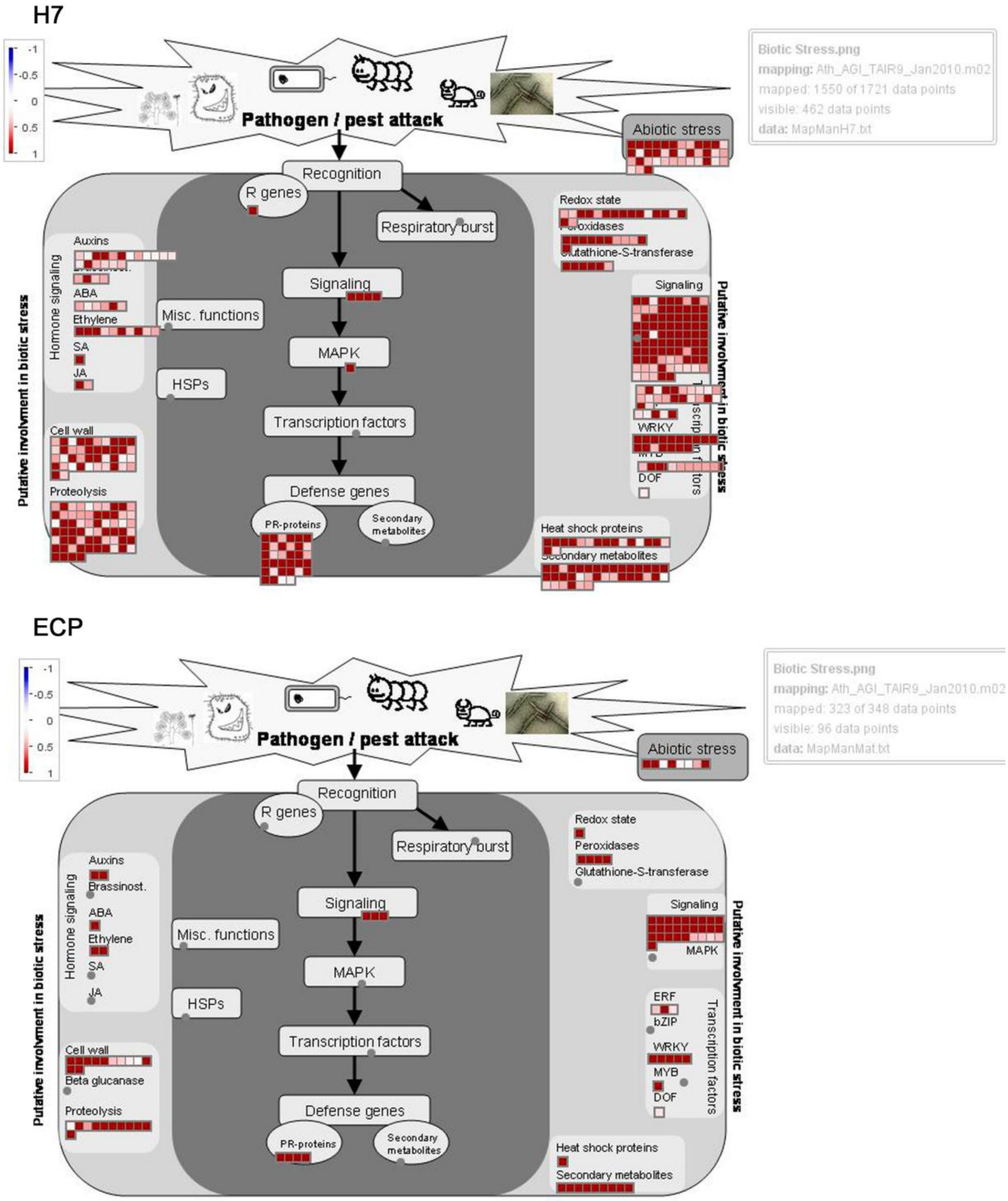

Figure S3. *A. thaliana* H7 flagella and ECP fimbriae responsive genes common to overall *A. thaliana* responses to biotic stress mapped by MapMan [36].
